# ‘Grumbling’ Midgut Volvulus in an Older Paediatric Patient With Congenital Solitary Kidney: A Case Report

**DOI:** 10.7759/cureus.49202

**Published:** 2023-11-21

**Authors:** Matthew K Emmerson, Moya Dawson

**Affiliations:** 1 Paediatric Emergency Medicine, Oxford University Hospitals NHS Foundation Trust, Oxford, GBR

**Keywords:** case report, paediatric general surgery, emergency department, congenital solitary kidney, midgut volvulus

## Abstract

Midgut volvulus is a life-threatening condition, with the majority of cases presenting before the first year of life. Congenital gastrointestinal abnormalities can be associated with midgut volvulus; however, similar associations have not been described with congenital renal abnormalities. Congenital solitary kidney (CSK) means that a child is born with only one functional kidney.

Here, we describe a case of a five-year-old child with CSK who atypically presents with midgut volvulus. The case highlights how midgut volvulus may present with chronic symptoms in those over the age of one and the importance of upper GI contrast studies for diagnosis of midgut volvulus in this population and suggests CSK along with other causes of solitary kidney as possible risk factors for volvulus.

## Introduction

Intestinal malrotation is a congenital abnormality resulting from the incomplete rotation of the foetal gut during weeks 4-10 of embryogenesis. Ninety percent of cases present before the first year of life with bilious vomiting and abdominal pain [1]. However, some patients are asymptomatic and are only diagnosed incidentally during surgery or at autopsy [2]. Midgut volvulus is a serious complication of intestinal malrotation which can result in bowel necrosis, obstruction and death [3].

Malrotation and volvulus have a strong association with congenital gastrointestinal abnormalities such as abdominal wall defects (gastroschisis and omphalocele), congenital diaphragmatic hernias and heterotaxic syndromes [4]. However to date, no relationship has been described with congenital abnormalities of the kidneys.

Congenital solitary kidney (CSK) is the anatomical or functional absence of one kidney from birth [5]. This can occur due to a failure of embryonic renal tissue formation (renal agenesis), or due to extreme forms of dysplasia (renal aplasia and multicystic dysplastic kidney) [5]. It is a relatively common occurrence with an incidence of 1 in 2000 to 1 in 4300 live births [6,7].

There have been several case reports of post-nephrectomy patients developing a caecal volvulus [8-10] and one report of a child developing malrotation with a horseshoe kidney [11]. Apart from this one case, no other cases have been reported of children being born with a solitary kidney and developing midgut volvulus after the age of one.

Here, we describe a case of intestinal malrotation with midgut volvulus in a five-year-old boy who was born with a solitary right kidney.

## Case presentation

A previously well five-year-old boy presented to A&E with severe abdominal pain around the umbilicus. The pain had started suddenly that morning after he had his usual cup of tea. After the pain started, he began to vomit clear, non-bilious, non-bloody fluid. He was able to drink water but was not eating. He had opened his bowels that morning, passing stool of normal colour and consistency for him.

Over the previous two years, he had had several episodes of similar upper abdominal pain. These could occur weekly or monthly and were unpredictable, although appeared to happen towards the end of the week. Usually, there was no vomiting with the pain and it settled within a few hours. He was completely well in-between episodes. His mother had taken him to the GP several times for this, but no investigations had yet taken place.

The child’s past medical history included being born with a single right kidney, which was discovered at three months of age when he had an episode of urosepsis. He has a regular annual follow-up appointment to check on his renal function. He also had eczema, for which he used emollients and mometasone steroid cream. He was on no other medication. He had no known allergies, his immunisations were up to date and there were no social concerns.

On examination, he was pale, afebrile and in a significant amount of pain, unable to stand up straight or lie fully extended. His abdomen was generally soft with no guarding. It was not distended but had a palpable fullness of the epigastrium. Both testes were present and mobile. Post-examination, the child vomited copious amounts of dark green bilious vomitus.

Investigations

**Figure 1:**
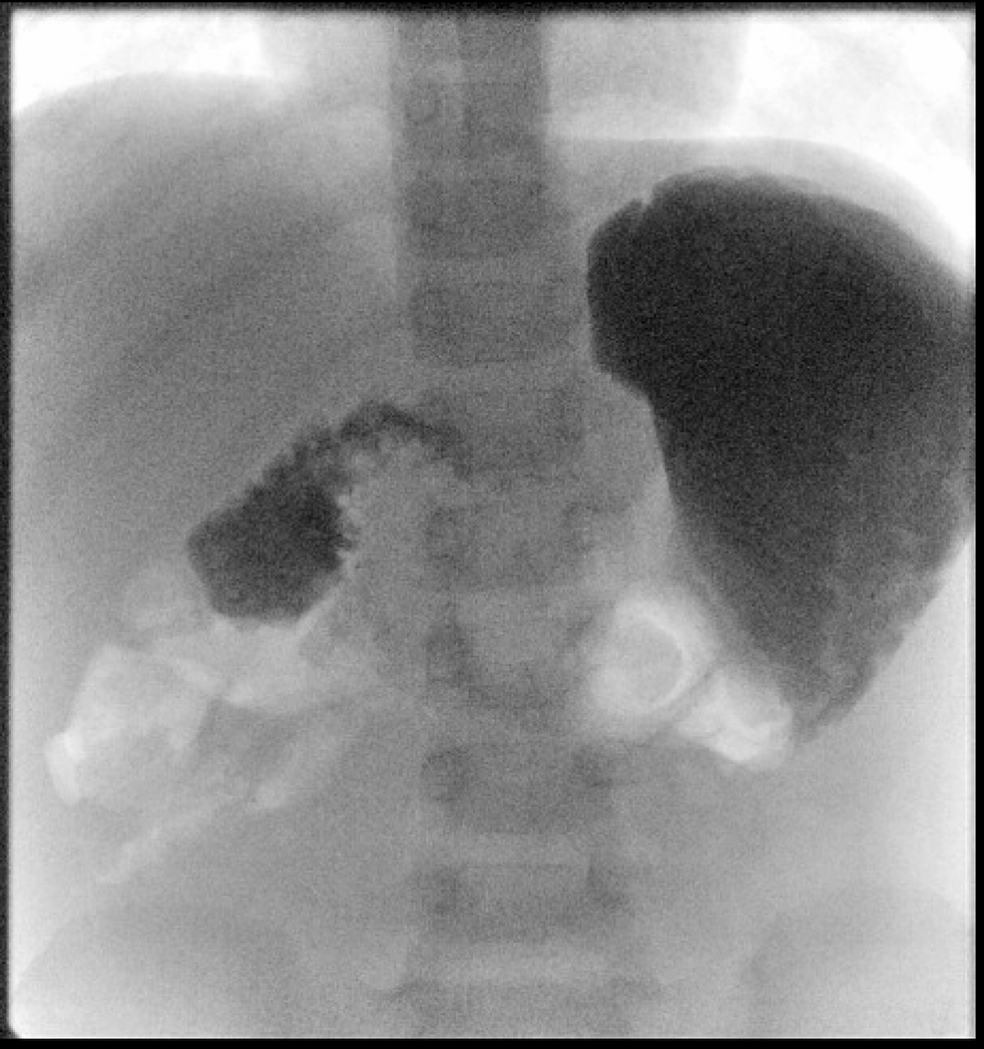
Water-soluble barium contrast X-ray revealing obstruction in the duodenum at D2

An initial point of care glucose demonstrated a blood glucose level of 10.4 mmol/ml and ketones of 0.4 mmol/L alongside a urine dip showing 2+ glucose. Subsequent laboratory findings showed normal glucose, ketones, amylase and lactate. Ultrasound was unable to identify the superior mesenteric artery and mesenteric vein due to epigastric gas obscuring the view. A water-soluble barium contrast revealed an obstruction in the duodenum at D2 (Figure 1), likely to be intestinal malrotation with midgut volvulus.

Differential diagnosis

Prior to the bilious emesis, the main differential diagnosis was of an atypical presentation of malrotation/volvulus, even though the longevity of the preceding intermittent symptoms caused some diagnostic uncertainty. Other differential diagnoses included pancreatitis, excluded by the normal amylase and early diabetes mellitus, also excluded by a subsequent normal glucose value and normal venous blood gas. Following the bilious emesis and upper GI contrast study results, malrotation with a possible volvulus was our working diagnosis.

Treatment and outcome

A successful laparotomy and Ladd's procedure were carried out the same evening with no complications. The bowel was found to be dusky with an oedematous mesentery but was not necrotic; reducing the volvulus allowed it to reperfuse successfully. The patient was discharged home four days later and was well at follow-up six months later.

## Discussion

Midgut volvulus is generally seen in infants below the age of one, with over 90% of cases occurring in this population [1]. In those above this age range, volvulus and intestinal malrotation can be mistaken for a range of conditions, such as pancreatitis, abdominal tuberculosis and gastro-oesophageal reflux, or even dismissed as a functional disorder [2]. This risk of misdiagnosis is exacerbated by an increased propensity for more atypical and chronic presentations of volvulus in the older population compared to those below the age of one [12,13]. Atypical presentations of volvulus most commonly include recurrent colicky abdominal pain and non-bilious vomiting [2]. As such, this case study helps highlight how important it is to consider volvulus as a differential even in patients outside of the classic age range and presentation.

When diagnosing volvulus, accurate and reliable imaging is key. The modalities of choice tend to be abdominal Doppler ultrasound, CT scan with contrast and upper GI contrast study. With abdominal ultrasound, malrotation can be identified through the ‘whirlpool’ sign describing the spiralling of the superior mesenteric vein and small intestine around the superior mesenteric artery [14]. A recent meta-analysis demonstrated a 94% sensitivity and 100% specificity of abdominal ultrasound for diagnosing volvulus [15]. However, not only is this modality highly dependent on radiographer technique and experience [15], but it is also not as accurate for diagnosing midgut volvulus presenting in patients older than one year due to the presence of bowel gas, as seen in this case study. Instead, upper GI contrast studies, involving anterior-posterior and lateral projections of the abdomen on barium swallow, or CT with contrast are preferred [16]. One study of 35 patients older than one year old showed that upper GI contrast studies diagnosed malrotation in all patients [2]. As such, in older children presenting with midgut volvulus, upper GI contrast studies or contrast abdominal CTs are the preferred imaging modalities.

The relationship between paediatric gastrointestinal problems and CSK is not one that has been investigated extensively. One systematic review found that CSK can be associated with other congenital abnormalities, most commonly anomalies of the kidneys and urinary tracts such as vesicoureteral reflex, with a prevalence of 32% in this paediatric population [6]. However, the same review found that 31% of patients had extra-renal abnormalities, 16% of which were gastrointestinal. In another review, anal atresia was found to be the most common gastrointestinal abnormality [17]. Given the correlation between these gastrointestinal anomalies and CSK, it would be interesting to investigate further whether CSK could be a potential risk factor for the development of late-onset midgut volvulus through a systematic review.

Only one other case study has described a patient with CSK (in this case, a horseshoe kidney) developing malrotation after the age of one [11]. However, several case studies have described patients who have undergone nephrectomies and then developed caecal volvulus above the age of one. These kidneys have been removed for a variety of reasons including Wilm’s tumour treatment [10], renal carcinoma treatment [9,18] and palliation [8]. In the majority of cases, the right kidney has been removed. Papers have hypothesised the volvulus may be due to the retroperitoneal approach taken to remove the kidney or that volvulus is simply a potential complication after intra-abdominal surgery [18]. However, given the observations from this case study of a patient who was born without a left kidney developing volvulus later in life, we hypothesise that the lack of renal tissue in the retroperitoneum may predispose patients to a higher risk of volvulus, with lack of a right kidney predisposing to caecal volvulus and lack of a left kidney to midgut volvulus due to their anatomical proximity to surrounding organs. Although extensive further investigations would be required to validate this superficial hypothesis, it does appear to hold for removal of other retroperitoneal organs such as the pancreas which can demonstrate gastric volvulus on removal [19,20].

## Conclusions

In patients outside of the classical age range of younger than one year old, midgut volvulus can present abnormally with chronic colicky abdominal pain and non-bilious vomiting. This can be confused with a range of differential diagnoses including pancreatitis, gastro-oesophageal reflux or a functional disorder. In this older patient cohort, a high index of suspicion along with an upper GI contrast study or CT abdomen with contrast is important in diagnosing midgut volvulus.

This case and other similar cases suggest that a solitary kidney, due to CSK or post-nephrectomy, may be a risk factor for volvulus development later in life. However, significantly more research is required to elucidate if this correlation is causal.
